# Lipid emulsion modulates myogenic and collagen‐related gene expression in skeletal muscle of preterm fetal sheep

**DOI:** 10.1113/EP093580

**Published:** 2026-04-09

**Authors:** Xinrui Li, Sarah M Alaniz, Samantha Louey, Jeanene Marie Deavila, Sonnet S. Jonker, Min Du

**Affiliations:** ^1^ Nutrigenomics and Growth Biology Laboratory, Department of Animal Sciences Washington State University Pullman Washington USA; ^2^ Center for Developmental Health Oregon Health & Science University Portland Oregon USA; ^3^ Lawrence D. Longo, MD Center for Perinatal Biology, Department of Basic Sciences Loma Linda University School of Medicine Loma Linda California USA

**Keywords:** fibrogenesis, Intralipid, myogenesis, preterm sheep, skeletal muscle

## Abstract

Although intravenous lipid emulsions are routinely administered to preterm infants, their specific effects on skeletal muscle development remain unclear. In this study, a soybean oil‐based lipid emulsion (Intralipid 20^®^) was administered via intravenous infusion to fetal sheep (gestational day 88–90) at a dose rising from 1 g/kg/day (day 0) to 3 g/kg/day (days 2–8). Intralipid infusion did not alter overall fetal body weight, tibialis anterior (TA) muscle mass or serum testosterone levels. Histological analyses revealed no significant differences in muscle fibre diameter or collagen content in TA muscles between groups. However, Intralipid significantly upregulated the expression of key myogenic regulatory genes, including *Myog* (myogenin) and *Myod* (myogenic differentiation 1), while downregulating the expression of several genes associated with fibrogenesis: *Col1a1* (collagen type I α1 chain), *Col3a1* (collagen type III α1 chain), *Lh2b* (lysyl hydroxylase 2b) and *P4ha* (prolyl 4‐hydroxylase α). In contrast, Intralipid had no significant effect on the expression of genes associated with intramuscular adipogenesis, including *Pparg* (peroxisome proliferator‐activated receptor γ), *Pdgfra* (platelet‐derived growth factor receptor α), *Zfp423* (zinc finger protein 423), *Slc27a1* (solute carrier family 27 member 1), *C/ebpa* (CCAAT/enhancer‐binding protein α) and *Fasn* (fatty acid synthase). Similarly, genes related to inflammation, such as *Tnfa* (tumour necrosis factor α), *Il‐6* (interleukin 6), *Tlr4* (Toll‐like receptor 4) and *Tlr2* (Toll‐like receptor 2), were unaffected. In conclusion, these findings indicate that short‐term lipid exposure alters gene expression patterns without measurable structural changes, suggesting that transcriptional responses may precede overt morphological remodelling in fetal skeletal muscle.

## Abstract (highlights)

- **What is the central question of this study?**
  How do intravenous lipid emulsions, which are commonly used in the clinical care of preterm infants, affect the development of skeletal muscle?
- **What is the main finding and its importance?**
  Intralipid infusion increases the expression of key myogenic regulatory genes while decreasing the expression of genes involved in collagen synthesis, without altering adipogenic or inflammatory markers. These findings suggest that lipid supplementation may alter the expression of genes related to muscle differentiation and fibrogenic signalling in skeletal muscle, providing insight into how nutrition shapes musculoskeletal pathways in preterm infants.

## 1 INTRODUCTION

Premature infants are separated from the in utero environment early, truncating a critical period of late gestation growth and development (Kugelman & Colin, 2013). As a result, they face substantial nutritional and metabolic challenges that can impair organ maturation (Rigo & Senterre, 2006). One result is premature birth‐induced persistent alterations in skeletal muscle development, characterized by myofibre atrophy, increased oxidative stress and chronic inflammation, which collectively contribute to diminished exercise capacity throughout the lifespan (Deprez et al., 2021).

Fetal muscle development is crucial because it determines the number and type of muscle fibres, which significantly influence postnatal growth and muscle function (Du et al., 2010). Proper skeletal muscle development relies on an adequate supply of nutrients, particularly the appropriate intake of protein and essential fatty acids (Micke et al., 2011; Silveira et al., 2008). Due to immature gastrointestinal systems, many premature infants must rely on parenteral nutrition to maintain energy and tissue growth (Darmaun et al., 2018).

Lipid emulsions are the primary energy source in parenteral nutrition for premature infants (Cleminson et al., 2021). Beyond providing high‐density calories, the fatty acids from these emulsions participate in membrane synthesis and act as signalling molecules that influence metabolic and inflammatory pathways (Cleminson et al., 2021). They also affect immune system maturation and lipid‐mediated signalling in skeletal muscle cells, thereby contributing to muscle fibre growth and differentiation (Frazer & Martin, 2021). In recent years, with continuous advancements in lipid emulsion formulation, such as soybean oil‐based emulsions, medium‐ and long‐chain triglyceride blends, and olive oil or fish oil‐enriched emulsions, their effects on body composition and muscle development in premature infants have garnered increasing attention (Calder et al., 2018; Wang et al., 2016). Polyunsaturated fatty acids affect skeletal muscle development by increasing muscle satellite cell activity (Risha et al., 2021), promoting protein anabolism through activation of mammalian target of rapamycin (mTOR)‐related pathways (Nwachukwu et al., 2017), and reducing inflammatory responses by regulating cytokine signalling (Jannas‐Vela et al., 2023; Nwachukwu et al., 2017). However, the effects of lipid emulsions on skeletal muscle development in premature infants and the underlying mechanisms remain unclear, and the optimal clinical application strategy remains to be established.

Fetal sheep are commonly used as a model for pregnancy studies (Morrison et al., 2018). Using pre‐term fetal sheep, this study aimed to explore the potential effects of lipid emulsions on skeletal muscle development in premature infants, providing a theoretical foundation for optimizing nutritional support strategies in this population. We hypothesized that intravenous lipid supplementation during late gestation would modulate the expression of genes associated with myogenic differentiation and fibrogenesis, potentially enhancing the myogenic regulatory programme and modulating fibrogenic signalling within the skeletal muscle of the preterm fetus.

## 2 METHODS

### 2.1 Ethical approval

All animal procedures and sample collections were conducted at Oregon Health & Science University (OHSU) and were approved by the OHSU Institutional Animal Care and Use Committee (IACUC protocol number 007). These experiments were performed in accordance with the guidelines laid down by the OHSU animal welfare committee and comply with the animal ethics policies.

### 2.2 Animal care and tissue collection

The preterm sheep model was established based on a previous study (Li et al., 2025), using surgical catheterization of fetal sheep from ewes carrying twins at 84–86 days of gestation. The experimental protocol began 4 days after surgery (at 88–90 days of gestation) and continued for 8 days until fetuses reached 96–98 days of gestational age. One twin (Intralipid group, *n* = 9, 6 female, 3 male) received a graduated intravenous administration of lipid emulsions (Intralipid 20^®^, Frensenius Kabi, Lake Zurich, IL, USA, cat. no. 831800311; soybean oil based), beginning at 1 g/kg/day on Day 0, increasing to 2 g/kg/day on Day 1, and maintained at 3 g/kg/day from Days 2–8, while its sibling served as a control (control group, *n* = 8, 2 female, 6 male) by receiving only Lactated Ringer's injection USP (Baxter Healthcare Corporation, Deerfield IL, USA, NDC 0338‐0117‐04) throughout the experimental period. Physiological data from this cohort were previously published (Barooni et al., 2025).

At the end of the experiment, ewes were humanely euthanized with an intravenous overdose of a commercial sodium pentobarbital solution. Following maternal euthanasia, fetuses were removed and their hearts arrested in diastole by intravenous administration of saturated potassium chloride via the umbilical vein. Tibialis anterior (TA) muscle was harvested and one portion was fixed in 4% paraformaldehyde, while the remaining tissue was wrapped in foil, snap‐frozen in liquid nitrogen, and stored at −80°C for subsequent biochemical analyses.

### 2.3 Enzyme‐linked immunosorbent assay

EDTA‐anticoagulated plasma (collected at the end of the experiment) testosterone levels were measured using the Testosterone Total ELISA kit (Eagle Biosciences, Amherst, NH, USA, cat. no. TST31‐K01) according to the manufacturer's instructions.

### 2.4 Haematoxylin–eosin staining

After 24 h of fixation, tissues were transferred to 70% ethyl alcohol for up to 5 days before processing and embedding in paraffin. Paraffin‐embedded tissue sections were cut to 7 µm thickness using a microtome (RM2255, Leica Biosystems, Nussloch, Germany), which then underwent a standardized histological preparation process beginning with xylene dewaxing followed by rehydration through a descending gradient of alcohol solutions. For haematoxylin–eosin (H&E) staining, nuclei were stained with haematoxylin and cytoplasmic components were counterstained with eosin. To preserve tissue architecture for long‐term storage, sections were processed through an ascending ethanol series for dehydration, cleared with xylene, and permanently mounted with sealant (Estrada et al., 2017). H&E staining was performed according to a previously published protocol (Chlipala et al., 2020). Microscopic images were captured using a Leica DM2000 microscope (Leica Microsystems, Wetzlar, Germany).

### 2.5 Measurement of fibre diameter

Muscle fibre diameters were measured as previously described (Qin et al., 2020). Briefly, measurements were made using ImageJ software developed by National Institute of Health (Bethesda, MD, USA) and the minimal Feret's diameter was recorded. For each lamb, approximately 300 myofibres from different sections were analysed, excluding fibre with incomplete sarcolemma, and the mean fibre diameter per animal was calculated for statistical comparison among groups.

### 2.6 Measurement of collagen content

Tissue samples were acid‐hydrolysed (6 M HCl at 95°C for 6 h) and the supernatant was used for quantifying hydroxyproline content using the Hydroxyproline Colorimetric Assay Kit (Elabscience, Wuhan, China, cat. no. E‐BC‐K062‐S). The collagen content was determined by multiplying hydroxyproline content by 7.46 (Colgrave et al., 2008).

### 2.7 Real‐time quantitative PCR analysis

Lyophilized samples were used for RNA extraction and reverse transcribed into cDNA using a reverse transcription kit (Bio‐Rad Laboratories, Hercules, CA, USA, cat. no. 1708891). RT‐qPCR was conducted by mixing Bio‐Rad SYBR Green (cat. no. 1725150), cDNA, and specific primers, and running the reaction on the Bio‐Rad CFX system under the following thermal cycling conditions: initial polymerase activation and DNA denaturation at 95°C for 3 min, followed by 40 amplification cycles consisting of denaturation at 95°C for 10 s and extension at 60°C for 30 s. A melt curve analysis was subsequently performed from 65°C to 95°C, with 0.5°C increments and a dwell time of 2 s per step. The relative mRNA expression levels were quantified using the $2^{-\Delta \Delta C_{t}}$ method (Livak & Schmittgen, 2001), with *Rpl13* as the reference gene, and the primer sequences are listed in the Table 1.

**TABLE 1:** Primer sequences used for RT‐qPCR analyses.

Gene name	Sequence (5′–3′)	
	Forward	Reverse
*Myog*	AATGAAGCCTTCGAGGCCC	CGCTCTATGTACTGGATGGCG
*Myod*	CTACGACCGCGCTTACTACA	CGCGTCGGCCAGTAGAA
*Myf5*	CCCACCTCAAGTTGCTCTGA	TGCTCTGAGTTGGTGATCCG
*Desmin*	GCGCAGGATCGAATCTCTCA	AGCTGTGAGGTCTGGTTTCG
*Col1a1*	TAAGGGTGACAGAGGCGATG	GGACCGCTAGGACCAGTTTC
*Col3a1*	CAAAGGAGAGCCAGGAGCAC	CTCCAGGCGAACCATCTTTG
*Lh2b*	ATGCCAATCAAGAGGATCTG	CAGGTAGCGTTTCCCAATGT
*P4ha*	GATAAGGCGCTTTTGCTCAC	ATCCACAGCAGCACCTTTTT
*Pdgfra*	CGAACTGAAGATGCACGGGA	CGAAGGGGTGCTGTATGGAA
*Zfp423*	ACACCATGAGCACCAAAAGC	AGGCACCAGCAACTTCTGGA
*Pparg*	ACGGGAAAGACGACAGACAAA	AAACTGACACCCCTGGAAGATG
*C/ebpa*	TGCTGGCTGCAAAAAGTATG	CCCTGTAGTGAAGGCAGAGC
*Slc27a1*	ACTGTCTGCCCCTGTACCAC	GGCTGGCTGAAAACTTCTTG
*Fasn*	CTTAACAGCACGTCCCCCAT	TCCTCGGGCTTGTCTTGTTC
*Tnfa*	ACACCATGAGCACCAAAAGC	AGGCACCAGCAACTTCTGGA
*Il‐6*	TCATCCTGAGAAGCCTTGAGA	TTTCTGACCAGAGGAGGGAAT
*Tlr4*	TGCTGGCTGCAAAAAGTATG	CCCTGTAGTGAAGGCAGAGC
*Tlr2*	CAAGAGGAAGCCCAGGAAG	TGGACCATGAGGTTCTCCA
*Rpl13*	AGTACCGCTCCAAACTTATC	TCCTTCTTATAGACGTTCCG

### 2.8 Statistical analysis

For all experiments, data were expressed as means ± SD. Statistical analysis was carried out using Student's *t*‐test to compare between groups, with calculations performed in GraphPad Prism 8 (GraphPad Software, San Diego, CA, USA). Statistical significance was established at *P* < 0.05 for all comparisons.

## 3 RESULTS

### 3.1 Effects of lipid infusion on skeletal muscle of preterm sheep

**FIGURE 1:**
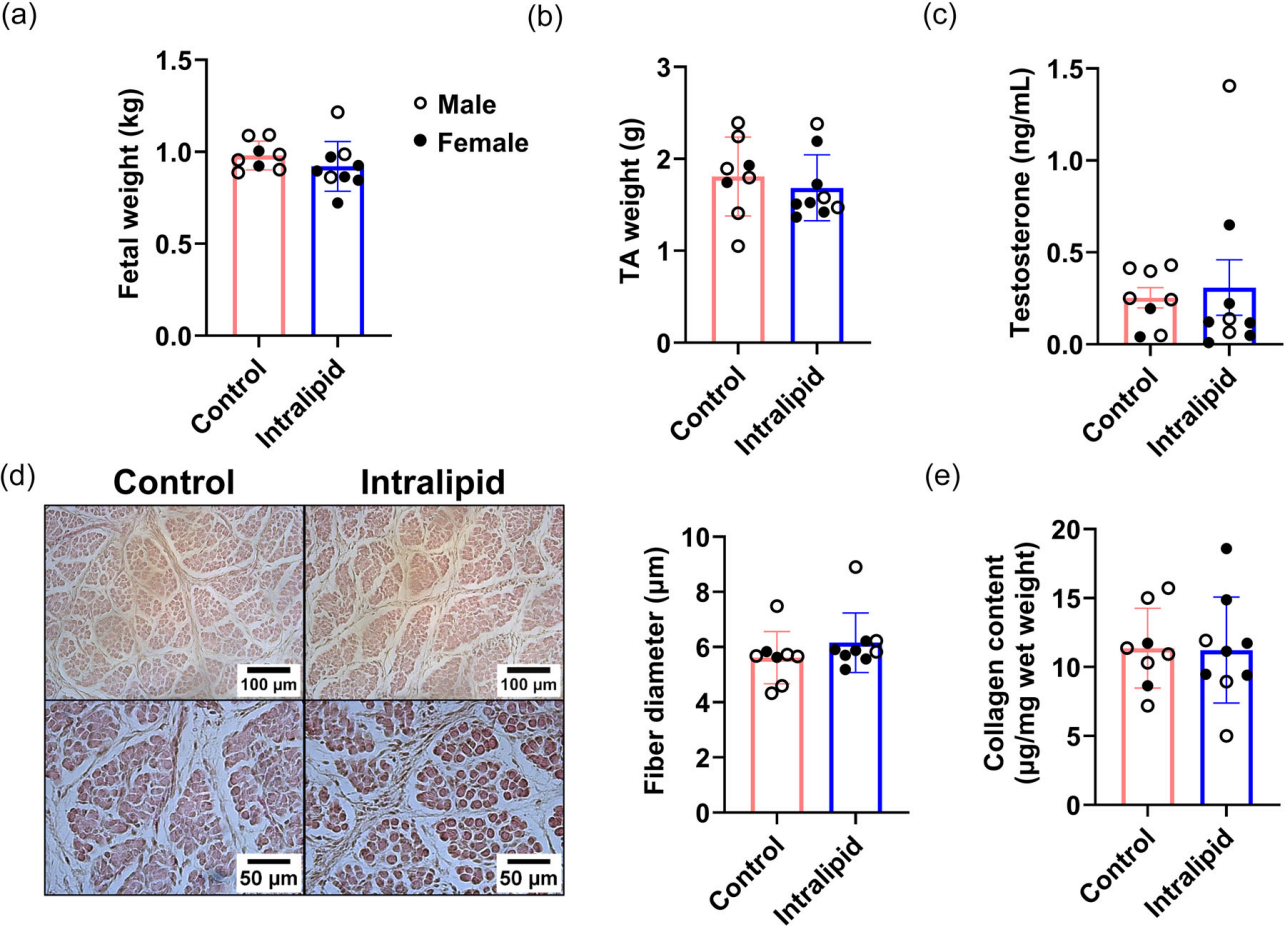
Effects of Intralipid infusion to preterm sheep on the phenotype of skeletal muscle. (a) The weight of fetuses. (b) The weight of TA muscle. (c) The plasma levels of testosterone. (d) Representative images of H&E staining for TA muscle (left) and quantification of fibre diameter (right). Left panels: scale bars represent 100 µm (top) and 50 µm (bottom). (e) Measurement of collagen contents of TA muscle. Control group: *n* = 2F, 6M; Intralipid‐treated group: *n* = 6F, 3M; data are presented as means ± SD with individual data points; *P < 0.05, **P < 0.01.

Intralipid infusion did not alter overall fetal weight (*P* = 0.149, Figure 1a), nor the TA muscle mass (*P* = 0.266, Figure 1b). No significant differences in serum testosterone levels were detected between the two groups (*P* = 0.372, Figure 1c). Histological examination using H&E staining at both ×20 and ×40 magnification revealed that fibre diameters in the TA muscle remained comparable between Intralipid‐treated and control groups (*P* = 0.146, Figure 1d). Collagen content of TA muscle did not alter following Intralipid treatment (*P* = 0.470, Figure 1e).

### 3.2 Lipid emulsion affects myogenic progression in the TA muscle of preterm sheep

**FIGURE 2:**
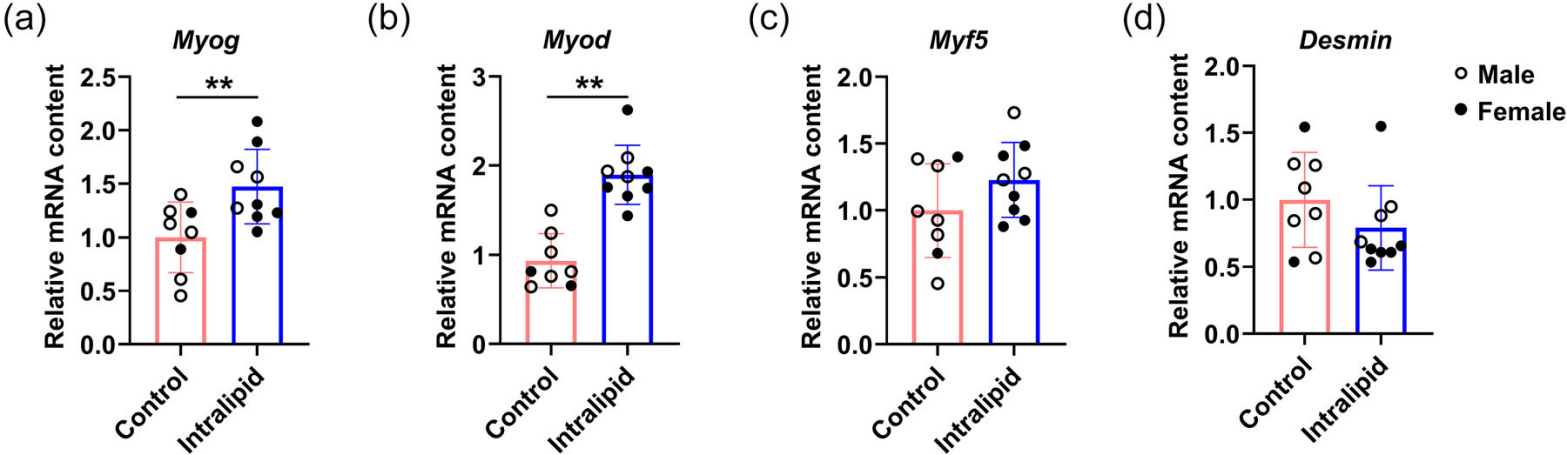
Intralipid infusion to pre‐term sheep enhanced TA muscle myogenesis. (a–d) Relative mRNA expression levels of *Myog*, *Myod*, *Myf5* and *Desmin*. Control group: *n* = 2F, 6M; Intralipid‐treated group: *n* = 6F, 3M; data are presented as means ± SD with individual data points; *P < 0.05, **P < 0.01.

Intralipid treatment enhanced the transcriptional activity of major myogenic regulators, with significant increases in both *Myog* (myogenin; *P* = 0.006, Figure 2a) and *Myod* (myogenic differentiation 1; *P* < 0.001, Figure 2b) mRNA expression, while expression levels of Myogenic factor 5 (*Myf5*), an early myogenic factor (*P* = 0.079, Figure 2c) and *Desmin*, a late myogenic factor (*P* = 0.108, Figure 2d), remained unchanged following treatment.

### 3.3 Lipid emulsion impairs collagen synthesis gene expression in the TA muscle of premature sheep

**FIGURE 3:**
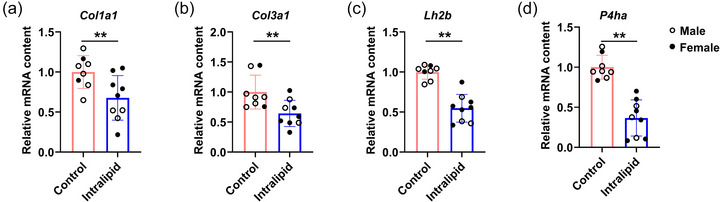
Intralipid infusion to pre‐term sheep reduced TA muscle collagen synthesis. (a–d) Relative mRNA expression levels of *Col1a1*, *Co3a1*, *Lh2b* and *P4ha*. Control group: *n* = 2F, 6M; Intralipid‐treated group: *n* = 6F, 3M; data are presented as means ± SD with individual data points; *P < 0.05, **P < 0.01.

Intralipid treatment significantly suppressed key genes involved in collagen synthesis, as shown by decreased mRNA expression levels of *Col1a1* (collagen type I α1 chain; *P* = 0.008, Figure 3a), *Col3a1* (collagen type III α1 chain; *P* = 0.005, Figure 3b), *Lh2b* (lysyl hydroxylase 2b; *P* < 0.001, Figure 3c) and *P4ha* (prolyl 4‐hydroxylase α; *P* < 0.001, Figure 3d) compared to controls. These genes encode major collagen components (COL1A1, COL3A1; Devos et al., 2023) and enzymes catalysing cross‐linking (LH2B, P4HA; Salo & Myllyharju, 2021).

### 3.4 Lipid emulsion has no effect on intramuscular fat development in the TA muscle of premature sheep

**FIGURE 4:**
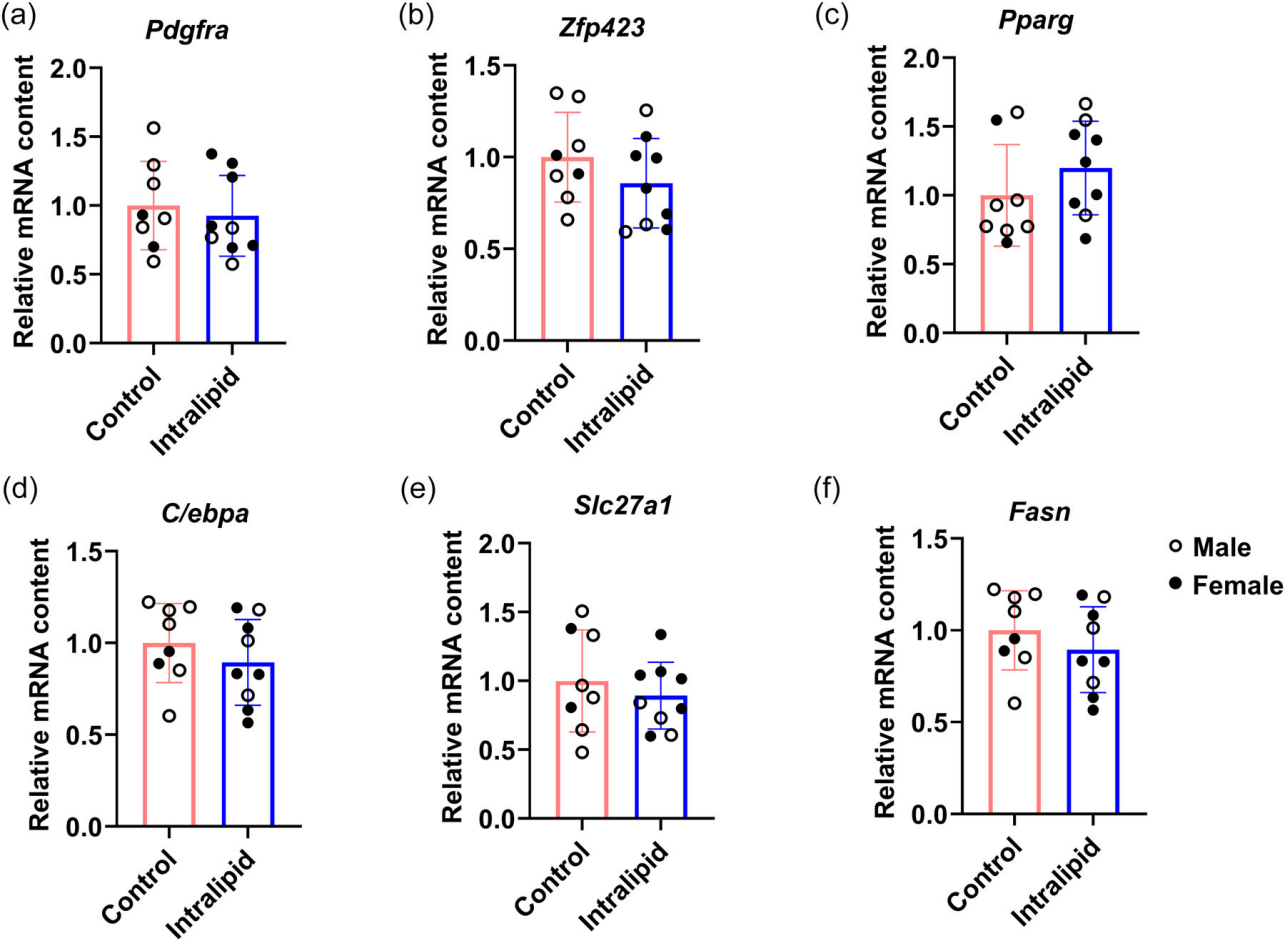
Intralipid treatment did not affect intramuscular adipogenesis in TA muscle of pre‐term sheep. (a–f) Relative mRNA expression of *Pdgfra*, *Zfp423*, *Pparg*, *C/ebpa*, *Slc27a1* and *Fasn*. Control group: *n* = 2F, 6M; Intralipid‐treated group: *n* = 6F, 3M; data are presented as means ± SD with individual data points; *P < 0.05, **P < 0.01.

Intralipid infusion had no detectable effect on intramuscular adipogenesis. *Pdgfra* (platelet‐derived growth factor receptor α; *P* = 0.312, Figure 4a), a marker of adipogenic progenitor cells (Dani & Pfeifer, 2017), did not differ between groups. Compared to controls, the expression of key transcription factors *Zfp423* (zinc finger protein 423; *P* = 0.125, Figure 4b), *Pparg* (peroxisome proliferator‐activated receptor γ; *P* = 0.132, Figure 4c) and *C/ebpa* (CCAAT/enhancer‐binding protein α; *P* = 0.175, Figure 4d) remained unchanged; these genes are essential for adipogenesis (Lee & Ge, 2014). Similarly, the expression levels of *Slc27a1* (solute carrier family 27 member 1; *P* = 0.245, Figure 4e) and *Fasn* (fatty acid synthase; *P* = 0.175, Figure 4f), which are involved in lipid synthesis and uptake during adipocyte differentiation (Zhang et al., 2021), showed no significant differences between groups.

### 3.5 Lipid emulsion had no effect on the inflammatory status of TA muscle in preterm sheep

**FIGURE 5:**
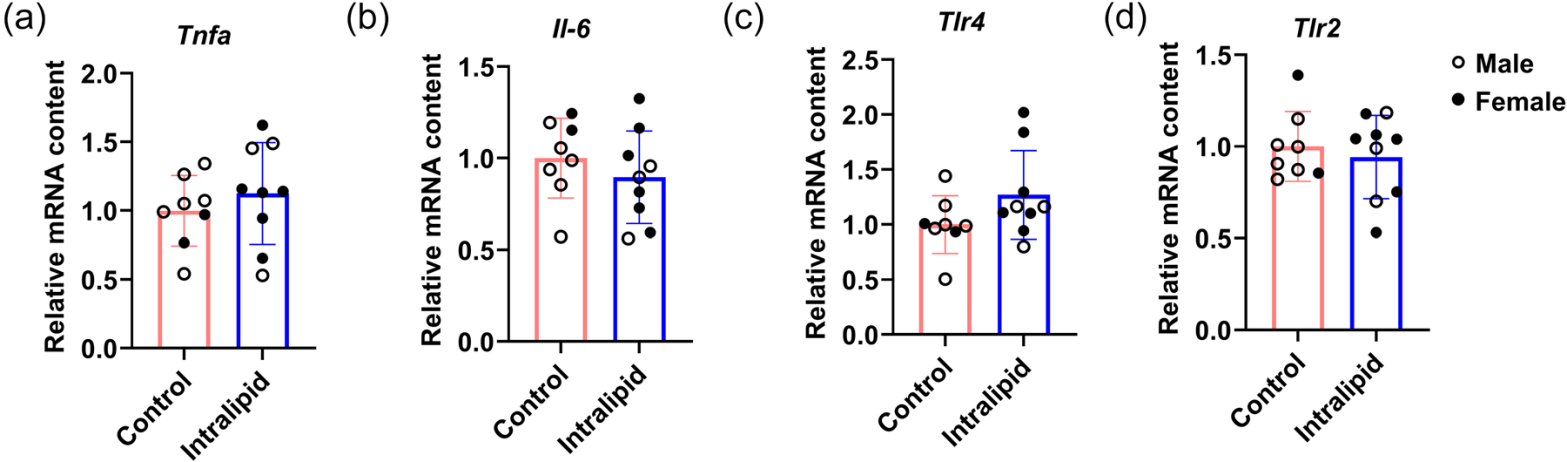
Intralipid infusion had no effect on the inflammatory status of TA muscle in pre‐term sheep. (a–d) Relative mRNA expression of *Tnfa*, *Il‐6*, *Tlr4* and *Tlr2* in TA muscle. Control group: *n* = 2F, 6M; Intralipid‐treated group: *n* = 6F, 3M; data are presented as means ± SD with individual data points; *P < 0.05, **P < 0.01.

Intralipid treatment had no effect on the expression of inflammatory cytokines *Tnfa* (tumour necrosis factor α; *P* = 0.220, Figure 5a; Popko et al., 2010) and *Il‐6* (interleukin 6; Choy & Rose‐John, 2017; *P* = 0.189, Figure 5b). The inflammatory receptors, *Tlr4* (Toll‐like receptor 4; *P* = 0.063, Figure 5c) and *Tlr2* (Toll‐like receptor 2; *P* = 0.290, Figure 5d), which respond to pathogens or tissue damage and trigger inflammatory responses (Mukherjee et al., 2016), remained comparable to controls.

## 4 DISCUSSION

In this study, in which Intralipid was infused into fetal sheep during mid‐gestation (88–98 days of gestation) to increase circulating lipids, we observed increased expression of some genes involved in skeletal myocyte differentiation as well as decreased collagen isoform expression. However, neither muscle weight nor morphology was changed. Further, lipogenesis‐mediating genes were unchanged in skeletal muscle. These data suggest that short‐term lipid supplementation might be insufficient to elicit anabolic or catabolic effects on fetal skeletal muscle.

### 4.1 Lipid availability influences myogenic gene expression without altering muscle structure

The observed increase in expression of mRNA in *Myod* and *Myog*, key regulators of myocyte differentiation (Vicente‐Garcia et al., 2022), may reflect transient myogenesis promoted by lipid emulsion. Elevated myogenic gene expression was not accompanied by increased muscle fibre diameter. This is consistent with previous studies showing that muscle development or early remodelling can occur without detectable changes in fibre diameter, particularly at early developmental stages (Moore et al., 2014), possibly due to the short treatment duration. The expression of *Myf5*, a gene involved in early myogenic commitment (Biressi et al., 2013), was not affected, suggesting that lipid emulsion did not significantly impact the initial specification of the myogenic lineage. Similarly, the stable expression of *Desmin*, encoding a key protein for maintaining the structural integrity of myofibre (Agnetti et al., 2022; Paulin & Li, 2004), indicates that lipid emulsion treatment exerted no effect on the late stage of myogenic differentiation. These findings indicate that while the fetal muscle responds to the presence of high circulating lipids, structural changes do not occur within a week.

### 4.2 Extracellular matrix‐related transcriptional changed do not translate to collagen remodelling

In contrast to the genes regulating muscle differentiation, expression of collagen metabolism‐related genes (*Col1a1*, *Col3a1*, *Lh2b*, and *P4ha*; Rong et al., 2019; Salo & Myllyharju, 2021) was downregulated due to Intralipid infusion, but the total collagen content remained stable. COL1A1 and COL3A1 are major components of skeletal muscle extracellular matrix (ECM; Chen et al., 2021), and moderate reduction of these collagens during early ECM remodelling is associated with a more permissive microenvironment for satellite cell migration and myogenic differentiation, partly due to decreased matrix stiffness (Chen & Li, 2009). However, the decrease in collagen metabolism‐related gene expression in this study was not accompanied by a reduction in skeletal muscle collagen content or an increase in myofibre diameter. Differences between gene expression and protein content in this context might be due to the short study timeline, as collagen has a very low turnover rate (Holwerda & van Loon, 2022).

It is important to acknowledge that the changes observed in this study are limited to the mRNA level. While mRNA expression often serves as a proxy for functional shifts, it does not always correlate with protein abundance due to post‐transcriptional regulatory mechanisms. For instance, myogenic factors myogenin (*Myog*) and myogenic differentiation 1 (*Myod*) are known to be regulated by specific microRNAs (e.g., *miR‐206*; Luo et al., 2013) and ubiquitin‐mediated protein degradation (Kudryashova et al., 2005), which can decouple transcript levels from protein activity. Similarly, collagen deposition is heavily dependent on post‐translational modifications and extracellular assembly. The lack of observed changes in muscle fibre diameter and collagen content may therefore reflect not only the short duration of the infusion but also a potential delay or attenuation in translating these transcriptional signals into functional protein remodelling.

### 4.3 Lipid supplementation does not activate adipogenic or lipogenic programmes in fetal muscle

The expression of lipogenesis‐related genes (*Pdgfra*, *Zfp423*, *Pparg*, *C/ebpa*, *Slc27a1* and *Fasn*) in mid‐gestation skeletal muscle are known to be upregulated during adipogenic differentiation and in response to lipid availability in adipose tissue (Li et al., 2025), but also exhibit dynamic developmental regulation during skeletal muscle development (Wang et al., 2024). *Zfp423* is developmentally regulated, controlling satellite cell fate and myogenic progression during regeneration (Addison et al., 2019), while *Pdgfra* marks fibro‐adipogenic progenitors (FAPs) that influence muscle repair and fat infiltration (Molina et al., 2021). Adipogenic regulators such as *Pparg*, *C/ebpa* and *Fasn* express in specific cell populations including FAPs, endothelial and myeloid cells in muscle, and contribute to intramuscular lipid metabolism, adipocyte formation and energy storage (Wang et al., 2024). Key lipogenic components like *Srebp1c* and *Fasn* are upregulated during muscle development and in response to metabolic cues, highlighting the coordinated control of lipid synthesis and skeletal muscle maturation (An et al., 2016). We therefore hypothesized that Intralipid infusion might alter their expression in fetal muscle. However, no significant changes were measured, in contrast to changes observed in adipose tissue (Li et al., 2025), liver and heart (Barooni et al., 2025). This pattern is consistent with the developmental stage of mid‐gestation skeletal muscle, in which adipogenic progenitors are present but remain largely quiescent (Zhao et al., 2023). After birth, and during growth toward weaning, intramuscular fat gradually emerges as resident preadipocytes which accumulate lipids, leading to the development of intramuscular adipocytes over time (McCoski et al., 2021). Thus, the absence of a transcriptional response in fetal muscle likely reflects the developmental immaturity of its adipogenic programme at this stage.

### 4.4 Inflammatory pathways remain unaffected by lipid emulsion exposure

Preterm individuals are often characterized by a heightened inflammatory profile, reflecting both a generally elevated inflammatory state and a greater vulnerability to inflammatory stimuli (Humberg et al., 2020). While soybean oil‐based fat emulsions (high in *n*‐6 polyunsaturated fatty acids) are known to promote inflammation (Bennett & Gilroy, 2016; Grimble, 2005; Hecker et al., 2015; Turner et al., 2016), our findings show that lipid emulsion administration did not significantly affect inflammation‐related markers in skeletal muscle of preterm sheep. This may suggest tissue‐specific inflammatory response in which skeletal muscle is protected (Galli & Calder, 2009). Nevertheless, it is important to caution about inflammation when administering these emulsions to preterm infants, because minimizing inflammation is crucial for preventing oxidative stress and other developmental complications (Martini et al., 2023).

### 4.5 Potential metabolic and signalling pathways linking lipids to transcriptional changes

High circulating lipids may influence the observed transcriptional changes in skeletal muscle through several non‐inflammatory mechanisms. Lipid availability can act as both an energy substrate and a signalling cue. Elevated plasma triglycerides and fatty acids may activate energy‐sensing pathways such as phosphoinositide 3‐kinase (PI3K)–Akt or AMP‐activated protein kinase (AMPK), which are known to regulate myogenesis in response to altered cellular energy status (Kjobsted et al., 2018; Park et al., 2014). Alternatively, lipids can exert direct signalling effects through fatty acid transporters and sensors, including CD36 and peroxisome proliferator‐activated receptor α pathways, which integrate lipid uptake with transcriptional control of myogenic and metabolic genes (El Ouali et al., 2023). Sterol regulatory element‐binding protein 1c, a master regulator of lipid metabolism, and carnitine palmitoyltransferase 1, involved in fatty acid oxidation, are also expressed in fetal skeletal muscle and may respond dynamically to circulating lipid levels by modulating gene networks related to differentiation and extracellular matrix remodelling (Defour et al., 2012; Watt & Hoy, 2012). Thus, increased lipid availability may transiently stimulate myogenic differentiation through metabolic–signalling crosstalk.

### 4.6 Potential endocrine influence of lipid infusion

In the fetal sheep model, systemic endocrine factors such as insulin and insulin‐like growth factors (IGF) are pivotal regulators of nutrient‐mediated skeletal muscle growth (Brown, 2014). While Intralipid provides a high‐density energy source that could theoretically stimulate these pathways, our previous physiological characterization of this specific cohort (Barooni et al., 2025) provides evidence that the observed effects are not secondary to systemic endocrine shifts. Specifically, while circulating IGF‐1 increased by 27% as a function of gestational age, these levels were not significantly altered by Intralipid treatment. Similarly, plasma IGF‐2 and insulin levels remained stable regardless of lipid infusion (Barooni et al., 2025). These findings suggest that the observed upregulation of *Myog* and *Myod* was likely driven by direct lipid‐mediated signalling or local paracrine mechanisms rather than a systemic endocrine response. The potential role of lipids as direct signalling cues – activating energy‐sensing pathways such as PI3K/Akt or AMPK – further supports the idea that the fetal muscle responds to metabolic cues independently of changes in the systemic IGF axis.

### 4.7 Limitations related to developmental stage and fetal sex

As a limitation, the sex of fetuses between treatments was not balanced, a common problem associated with fetal sheep studies due to the random assignment to each study group without prior knowledge of fetal sex. Testosterone is known to stimulate skeletal muscle growth (Kraemer et al., 2020), but no difference was detected between the two groups, which could be explained by the immaturity of fetuses at this stage. In addition, testosterone level was not affected by lipid infusion, suggesting that androgenic signalling did not contribute to the observed transcriptional changes. Given that testosterone can enhance myogenic differentiation through the Akt–mTOR pathway (White et al., 2013), the lack of difference indicates that the effects on muscle gene expression were more likely driven by direct lipid or energy‐dependent mechanisms rather than endocrine modulation.

### 4.8 Conclusion

Short‐term Intralipid infusion during mid‐gestation induced modest transcriptional changes in fetal skeletal muscle, including increased expression of myogenic genes and reduced expression of ECM‐related genes, while muscle structure, collagen content, lipid‐metabolic pathways and inflammatory markers remained unchanged. These findings suggest that while fetal muscle can sense increased lipid availability, transcriptional responses related to structural or metabolic remodelling appear to be limited at this developmental stage. Further work is needed to determine whether longer exposure or later developmental windows can more effectively influence muscle development in preterm infants.

## AUTHOR CONTRIBUTIONS

Xinrui Li, Sarah M Alaniz and Samantha Louey conceived and designed research; Xinrui Li, Jeanene Marie Deavila and Min Du performed investigation; Xinrui Li and Jeanene Marie Deavila analysed data; Xinrui Li, Min Du and Sonnet S. Jonker interpreted results; Xinrui Li and Min Du prepared figures; Xinrui Li drafted manuscript; Samantha Louey, Sonnet S. Jonker. and Min Du edited and revised manuscript. All authors have read and approved the final version of this manuscript and agree to be accountable for all aspects of the work in ensuring that questions related to the accuracy or integrity of any part of the work are appropriately investigated and resolved. All persons designated as authors qualify for authorship, and all those who qualify for authorship are listed.

## CONFLICT OF INTEREST

No conflicts of interest, financial or otherwise, are declared by the authors.

## Data Availability

Data will be made available upon reasonable request.
